# TEE-Graph: efficient privacy and ownership protection for cloud-based graph spectral analysis

**DOI:** 10.3389/fdata.2023.1296469

**Published:** 2023-11-30

**Authors:** A. K. M. Mubashwir Alam, Keke Chen

**Affiliations:** TAIC Lab, Computer Science, Marquette University, Milwaukee, WI, United States

**Keywords:** TEE, SGX, big graph, graph analytics, access pattern, ownership protection

## Abstract

**Introduction:**

Big graphs like social network user interactions and customer rating matrices require significant computing resources to maintain. Data owners are now using public cloud resources for storage and computing elasticity. However, existing solutions do not fully address the privacy and ownership protection needs of the key involved parties: data contributors and the data owner who collects data from contributors.

**Methods:**

We propose a Trusted Execution Environment (TEE) based solution: TEE-Graph for graph spectral analysis of outsourced graphs in the cloud. TEEs are new CPU features that can enable much more efficient confidential computing solutions than traditional software-based cryptographic ones. Our approach has several unique contributions compared to existing confidential graph analysis approaches. (1) It utilizes the unique TEE properties to ensure contributors' new privacy needs, e.g., the right of revocation for shared data. (2) It implements efficient access-pattern protection with a differentially private data encoding method. And (3) it implements TEE-based special analysis algorithms: the Lanczos method and the Nystrom method for efficiently handling big graphs and protecting confidentiality from compromised cloud providers.

**Results:**

The TEE-Graph approach is much more efficient than software crypto approaches and also immune to access-pattern-based attacks. Compared with the best-known software crypto approach for graph spectral analysis, PrivateGraph, we have seen that TEE-Graph has 10^3^−10^5^ times lower computation, storage, and communication costs. Furthermore, the proposed access-pattern protection method incurs only about 10%-25% of the overall computation cost.

**Discussion:**

Our experimentation showed that TEE-Graph performs significantly better and has lower costs than typical software approaches. It also addresses the unique ownership and access-pattern issues that other TEE-related graph analytics approaches have not sufficiently studied. The proposed approach can be extended to other graph analytics problems with strong ownership and access-pattern protection.

## 1 Introduction

Graphs are widely used in many domains, e.g., modeling social networks, knowledge graphs, and biological regulation networks (Backstrom et al., 2006; Chakrabarti and Faloutsos, 2006; Butenko et al., 2009; Sheth et al., 2019). These graph data are often large and sparse and may involve expensive analytics algorithms, such as graph spectral analysis (Ng et al., 2001; Newman, 2013), which require extensive computing resources.

With the wide deployment of public clouds, cloud-based solutions have been the top choice for most graph-data owners due to the storage and computational elasticity, which, however, raises several concerns. First, such graph data often involve sensitive personal information, e.g., social interactions. Data contributors have to trust that data owners can protect their privacy well when using cloud resources. Second, data owners want to maintain the confidentiality and ownership of their proprietary data as they collect the data with significant costs and hold the responsibility of protecting contributors' privacy. More recently, privacy laws such as GDPR (Chander et al., 2020; Zaeem and Barber, 2020) also guarantee fine-grained privacy rights, e.g., contributors can withdraw their data from sharing at any time: the right of revocation. These challenges raise the standard for cloud-based graph analytics solutions, which have not been comprehensively addressed by any existing studies yet (Plimpton and Devine, 2011; Meng et al., 2015; Shaon et al., 2017; Sharma et al., 2019; Sheth et al., 2019; Du et al., 2023).

To enable secure processing on untrusted platforms, researchers have been experimenting with novel crypto approaches, such as homomorphic encryption (HE) (Brakerski and Vaikuntanathan, 2011) and secure multi-party computation (SMC) (Huang et al., 2011; Mohassel and Zhang, 2017). In particular, Sharma et al. (2019) used novel protocol designs for graph spectral analysis which can be implemented with additive homomorphic encryption (Paillier, 1999) or somewhat homomorphic encryption (Brakerski and Vaikuntanathan, 2011). More recent advances in hybrid protocols (Mohassel and Zhang, 2017; Sharma and Chen, 2019) strive to reduce the overall costs of the frameworks by blending multiple crypto primitives to implement algorithmic components. However, all these pure software-based cryptographic solutions are still too expensive to be practical for most applications. As we will show, they often take magnitudes of higher costs than our proposed approach.

Recently, the trusted execution environment (TEE) has emerged as a more efficient approach to addressing secure outsourced computation performance and usability issues. It provides hardware support to create an isolated environment within a potentially compromised cloud server, where the entire system software stack, including the operating system and hypervisor, can be compromised. TEEs enable the concept of secure *enclaves*, which depends on hardware-assisted mechanisms to preserve the confidentiality and integrity of enclave memory. Users can pass encrypted data into the enclave, decrypt it, compute with plaintext data, encrypt the result, and return it to untrusted cloud components. TEEs have been available on many commodity CPUs and supported by public clouds. Intel Software Guard Extension (SGX) (Costan and Devadas, 2016) has been available in most Intel CPUs since 2015 and moved to server CPUs since 2022 (Intel, 2023). AMD secure encrypted virtualization (SEV) has been available in EPYC server CPUs since 2016. TEE-enabled servers are available in public cloud services: Microsoft Azure (Pietervanhove, 2023) and Alibaba (Alibaba, 2023) have provided SGX-enabled servers, and Google has adopted AMD SEV servers.

A few TEE-based studies have focused on graph data analysis problems (Shaon et al., 2017; Du et al., 2023). SGX-BigMatrix (Shaon et al., 2017) provides a general SGX-based matrix computation framework that can perform graph analytics with protected access patterns. It aims to reduce the difficulties for developers to handle TEE-specific programming and access-pattern protection. However, it does not address unique access-pattern issues with sparse large graphs. In practice, large graphs are often sparse, where sparse-matrix-based graph algorithms, e.g., spectral analysis on sparse matrices, might be used to achieve good performance. Meanwhile, a sparse matrix stores the entries with their indices, e.g., (*i, j, v*), where (*i, j*) is the index. Accessing by the index exposes sensitive information, e.g., the specific edge in the graph. Section 4.3 discusses the details of the problem. Thus, processing sparse graphs involves an intricate trade-off between privacy and performance.

Furthermore, most existing studies involve the data owner and cloud provider only, where the data owner fully represents the contributors, entirely ignoring the data contributors' ownership rights. They do not meet the new demands, e.g., guaranteeing the contributors' right to revoke the sharing of private data.

### 1.1 Scope of our research

**Figure 1:**
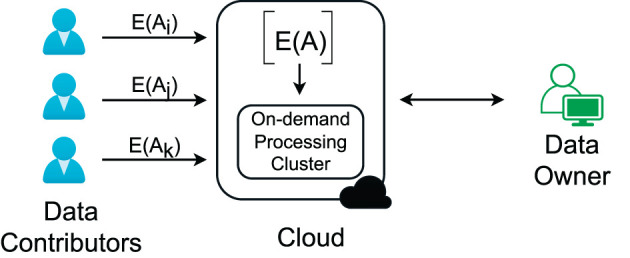
A more practical framework for outsourced graph analysis.

To address the above problems, efficient processing of large graphs, access-pattern protection, and contributors' ownership, we develop an approach based on a more practical cloud-centric framework for graph analysis. We start with an example where a social network service provider (data owner) collects users' (data contributors') interactions to understand social communities (see Figure 1). The users have the right to invoke the sharing of their data at any time. The service provider uses the cloud to store the interaction data and needs to preserve the data confidentiality at rest and in processing to fulfill its responsibility for preserving users' privacy and protecting its right to use the proprietary data.

The proposed approach will focus on complex graph analysis algorithms, i.e., graph spectral analysis, to show the unique advantages. Graph spectral analysis has numerous uses, including network partitioning (Newman, 2013), spectral clustering (Fowlkes et al., 2004), and web ranking (Berkhin, 2005). The specific analytics problem is challenging to handle due to the high complexity *O*(*N*^3^) for the complete solution and *O*(*kN*^2^) for top-k approximate solutions, where *N* is the number of nodes. Furthermore, the fundamental operation of the analysis task, eigendecomposition of large matrices, has even more extensive applications, such as dimensionality reduction (Jolliffe, 1986) and kernel-based learning methods (Scholkopf and Smola, 2002).

Within the cloud-based framework, the three parties collaborate to effectively collect and mine the graph data. Data contributors are willing to share their sensitive data with the data owner, who agrees to protect their data privacy^1^ and ownership. Contributors also request the ability to revoke access to their data at any point in time, e.g., a right guaranteed by certain privacy regulations such as GDPR, but still worry about the data owner may use their data even after the revocation. Meanwhile, the data owner uses public cloud resources to manage and mine the growing amount of contributors' data. However, the data owner does not trust the cloud provider can ensure data privacy and ownership.

Our proposed approach has been tailored to meet the above practical setup. (1) We use a TEE-based submission service to seal the contributor submitted data, which can be revoked on demand by the contributor. It also prevents data owner from seeing plaintext data and copying it for other unauthorized uses, e.g., selling data for profit. (2) We adopt a differentially private data encoding method to prevent inference attacks during data submission and access-pattern based attacks during spectral analysis. We study two approximate algorithms for confidential graph spectral analysis: the Lanczos method (Cullum and Willoughby, 1985) and the Nyström method (Fowlkes et al., 2004) for sparse graph data. The result will be compared with our previous developed pure-software cryptographic approach: PrivateGraph (Sharma et al., 2019).

In summary, our research has made four unique contributions:

- We provided strong ownership and confidentiality protection for both data contributors and owners, under the assumption of a compromised cloud server.
- We addressed the challenges for conducting spectral analysis on big graphs with TEE and design the TEE-Graph framework. It requires small trusted memory consumption and guarantees integrity protection of the graph.
- We meticulously analyzed the entire TEE-Graph framework and identify potential access-pattern-based side channel information during data submission and computation in TEE. Then, we develop a differentially private graph encoding method to protect privacy during graph submission and efficiently hide sensitive access patterns during spectral analysis with fully preserved data utility.
- Finally, we implemented TEE-Graph and measure cost reductions for the three involved parties. Our method performs 6000 × to 150,000 × faster than the baseline crypto approaches in PrivateGraph.

In the remaining sections, we will first present the background knowledge for our approach (Section 2), then we describe architecture of TEE graph (Section 3.2, dive in the technical details of the proposed approach (Section 4), discuss the evaluation result (Section 5.1), and, finally, give the closely related work (Section 6).

## 2 Preliminaries

This section will give the background knowledge about approximate spectral analysis algorithms for large graphs, a brief description of trusted execution environment, and differential privacy.

### 2.1 Graph spectral analysis

The core operation of graph spectral analysis is the eigendecomposition of the graph matrix, which yields eigenvalues and corresponding eigenvectors. Eigenvalues and eigenvectors provide valuable information about the structure of the graph matrix and have been used in many data mining algorithms, such as social community detection (Newman, 2013), spectral clustering (Fowlkes et al., 2004), web ranking (Berkhin, 2005), dimensionality reduction (Jolliffe, 1986), and kernel-based methods (Scholkopf and Smola, 2002). Specifically, for the graph adjacency matrix *W* of a *N*-node undirected graph, its normalized Laplacian matrix is defined as *L* = *I*−*D*^−1^*W*, where *I* is the *N*-dimensional identify matrix and *D* is the diagonal degree matrix, i.e., the only non-zero elements *D*_*ii*_ are the node *i*'s degree. We want to find the decomposition *L* = *UΛU*^*T*^, where the matrix *U* consists of the eigenvectors and Λ is a diagonal matrix with eigenvalues on the diagonal.

A complete eigendecomposition of a *N* × *N* matrix possesses a remarkable time complexity of *O*(*N*^3^). Hence, approximate algorithms are often used for big *N*, including the power-iteration Lanczos (Cullum and Willoughby, 1985) and matrix-sampling based Nyström methods (Fowlkes et al., 2004). These algorithms reduce the cost to *O*(*kN*^2^), *k*≪*N* and return only the top-k eigenvectors and values. The core and most expensive operation in these algorithms are matrix-vector multiplication (for power-iteration methods) and small matrix-matrix multiplication (for sampling methods). See Algorithms 1, 2 for the fundamental steps of Lanczos and Nyström methods, respectively. These algorithms reduce the complexity with some sacrifice in accuracy. A larger number of Lanczos iterations or a larger sampling rate for Nyström accounts for better accuracy, which however, increase the computational cost.

**Algorithm 1:**
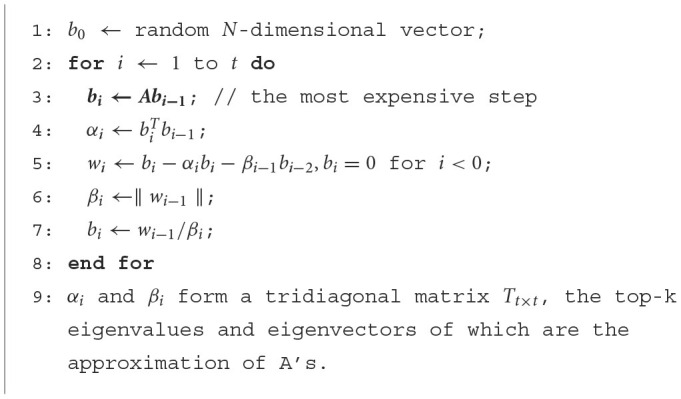
Lanczos method.

**Algorithm 2:**
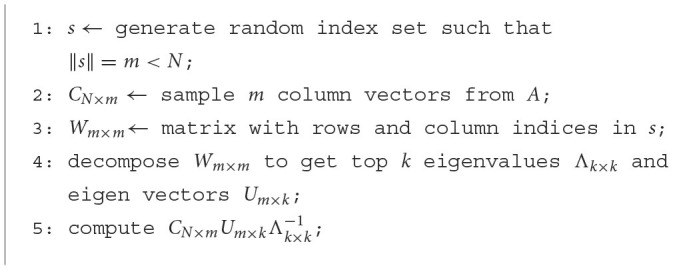
Nyström method.

### 2.2 Trusted execution environment

Trusted execution environment (TEE) is a hardware-based solution for executing code in a secure environment where powerful adversaries cannot access code or data within this secure area. Using TEEs, a user can run their sensitive computations in the secure area called *Enclave*, which uses a hardware-assisted mechanism to preserve the privacy and integrity of enclave memory. With TEEs, users can pass encrypted data into the Enclave, decrypt it, compute with plaintext data, encrypt the result, and return it to the untrusted cloud components. TEEs isolate private reserved memory for secure applications from other system components, such as operating systems and hypervisors. Thus, a powerful adversary controlling operating systems or hypervisors cannot breach TEEs.

The remote attestation procedure establishes the trust between the TEE hardware and the user via the CPU manufacturer's attestation server. The remote user must verify the correctness of the cloud hardware and the user binary to trust a TEE claimed by the cloud provider. Using remote attestation, the user can verify if the cloud provider uses certified TEE hardware and if the program running in an enclave is from a digitally signed binary.

Major cloud platforms have provided different types of TEE-enabled servers. Intel SGX is one of the popular TEE implementations. Since 2015, SGX has been available in most Intel CPUs. Similarly, ARM has TrustZone, and AMD has secure encrypted virtualization (SEV).

While all TEE implementations feature complete memory isolations from the system components and remote attestation to establish trust, they still suffer from side-channel attacks. Passive adversaries can exploit some attacks (Cash et al., 2015; Russakovsky et al., 2015; Zheng et al., 2017) by only observing interactions between TEEs and other system components. Some can even retrieve plaintext information directly from the Enclave via side-channel attacks. Based on the attack strategies, these attacks can be categorized as (i) memory/cache-targeted and (ii) microarchitecture-level attacks. In memory/cache-targeted attacks, the attacker exploits the interactions between TEEs and untrusted memory or applications and observes enclave memory page loading and CPU cache usages. Microarchitecture-level attacks utilize modern CPU features, such as CPU transient memory execution (Bulck et al., 2018), to retrieve fine-grained information from the low-level cache lines. An important approach to addressing the side-channel attacks is disguising access patterns known as data-oblivious algorithms (Alam and Chen, 2023).

In this study, we use TEE to address the high costs associated with the pure-software cryptographical approaches and use *differential privacy* to disguise the access patterns in TEE-based spectral analysis algorithms.

### 2.3 Differential privacy

Differential privacy (Dwork, 2006) is a standard notion in data privacy, which protects individual's privacy from inference attacks. For two datasets *A*_1_ and *A*_2_ that differ in exactly one record, let *M*(*A*_*i*_) be the mechanism that outputs noisy statistics *r*∈*R* of the datasets, then ϵ-differential privacy is satisfied if the following condition holds:


$$\begin{array}{l} Pr\left[M\left(A_{1}\right)=r\right]<=\exp\left(\epsilon \right)Pr\left[M\left(A_{2}\right)=r\right], \end{array}$$

where ϵ is the privacy parameter—the smaller it is, the better the preserved privacy. The basic idea is that with or without a victim present in the dataset, and the attacker cannot infer any useful private information from the noisy statistics *M*() about the victim. The mechanism *M* is defined as the additive perturbation of a specific query function *f*(*x*) that returns aggregate information of the dataset, such as the COUNT function: *M*(*A*) = *f*(*A*)+ random noise. The noise in the output is engineered to approximately preserve the utility of the query function while preventing attackers from inferring useful private information about any individual records in the database. Laplacian noise is one of the popular choices, where a noise is drawn from the Laplace distribution *Lap*(0, *b*), the density function of which is $\frac{1}{2b}\exp\left(-\frac{\left|x\right|}{b}\right)$. The parameter *b* is determined by the user-specified parameter ϵ and the sensitivity of query function: Δ = max|*f*(*A*_1_)−*f*(*A*_2_)| for any pair of *A*_1_ and *A*_2_ and *b* = Δ/ϵ. For example, the COUNT function has the sensitivity Δ = 1, and thus, the parameter *b* is set to 1/ϵ.

## 3 Privacy and ownership preserving graph analysis in the cloud: the architecture

We will first present the specific threat model that considers more fine-grained confidentiality and ownership protection. Then, we will present a TEE-based framework for confidential graph spectral analysis in the cloud that receives encrypted data from data contributors and conducts confidential analysis with TEE. Finally, we discuss the technical challenges under the threat model.

### 3.1 Threat model

The cloud-based framework comprises three parties: (i) the data owner who owns and analyzes data in the cloud, (ii) data contributors who agree to the data owner's privacy terms and provide their private data via data owner's data submission services, and (iii) the cloud provider who offers computation and storage services. Figure 1 shows the basic setting of the framework.

**Figure 2:**
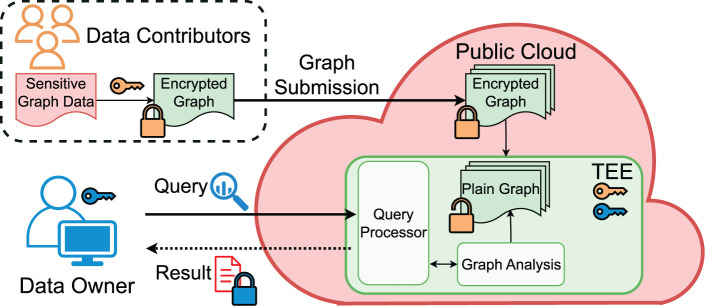
High-level overview of TEE-Graph architecture.

Different from software crypto approaches, we can upgrade the threat model with a compromised, possibly malicious cloud provider thanks to the cloud TEE, for which we will address both the confidentiality and integrity issues while leaving out the availability issue. Figure 2 illustrates our TEE-based framework. To guarantee the GDPR-level user privacy rights, we ensure the data owner's data confidentiality (only accessible to the authorized data owner) and the right of revocation. The data remain encrypted at rest, and without dedicated TEE programs, no other use of the data will be possible. Once a contributor revokes the sharing, the data owner cannot use them sneakily, e.g., by making a copy of the data.

#### 3.1.1 Assumptions about TEE

**Figure 3:**
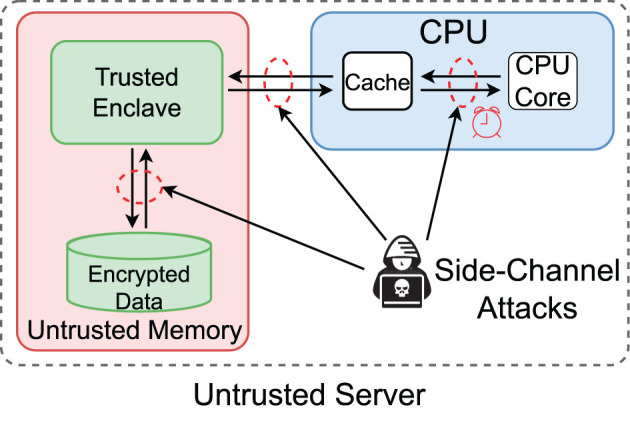
TEE, memory interactions, and side-channel vulnerabilities.

The TEE infrastructure (Costan and Devadas, 2016) provides the basic protection mechanism for the integrity and confidentiality of the data and programs in the enclave. Some TEEs, e.g., Intel SGX, have strong restrictions to achieve desired security. For example, the enclave program in the protected enclave memory area cannot access the file system APIs directly as the OS is not trusted. Thus, the encrypted data must be loaded from the main (untrusted) memory and then passed to the enclave. While adversaries cannot directly access the enclave, they can still glean information via side channels, such as memory access patterns and CPU caches. However, since cache-based attacks target all CPUs (regardless of having TEEs or not), we have to depend on manufacturers' microarchitecture level fixes. In contrast, the exposure of memory access patterns is inevitable as enclaves have to interact with the untrusted memory area, which is the main target we aim to protect. Figure 3 illustrates the TEE-specific threats.

#### 3.1.2 Compromised cloud provider

The cloud provider hosts the TEE infrastructure and allows remote attestation to verify the correctness of the TEE. Similar to other TEE-enhanced services, the cloud provider's memory is divided into the *Trusted Memory*, protected by the TEE, and the *Untrusted Memory* areas. The Cloud Provider has direct access to all code and data in the disk and the untrusted memory. Thus, data have to be encrypted in these areas. However, encryption does not prevent exposing data statistics, e.g., data size and read/write access patterns. The cloud provider cannot access trusted memory directly. However, since it controls the operating system and hypervisor, a compromised cloud provider may deploy system-privilege code in the untrusted memory to explore side channels of the TEE. It can also tamper with the data and program running in the untrusted memory and force the generation of page faults for enclave pages (Xu et al., 2015; Shinde et al., 2016).

#### 3.1.3 Dishonest data owners

Honest data owners will follow the contract with the contributors to use the data only for designated purposes. A dishonest data owner may try to make a copy of the data to circumvent contributors' revocation of sharing.

#### 3.1.4 Protected assets

We aim to ensure privacy and ownership guarantees for contributors and the data owner. First, we aim to protect the privacy rights of data contributors. Privacy laws such as GDPR require that the data owner protects data privacy, and the data contributor should have the right of revocation, i.e., stop the sharing with the data owner at any time. A dishonest data owner should not be able to continue to use the revoked data. Second, data owners are responsible for preserving contributors' data from adversaries, e.g., a compromised cloud provider, in collecting and processing data. Graph privacy includes edge privacy, e.g., whether an edge exists, and node privacy, e.g., the node degree and the k-hop neighborhood subgraphs.

#### 3.1.5 Scope of side-channel attacks

We consider only the memory side channels, which can be protected via protecting programs' access patterns. We assume although a cloud infrastructure can be compromised, the attacker cannot physically access the machine, e.g., by attaching a device to the server or touching the motherboard. Other side-channel attacks utilizing the unique features of cache or microarchitecture design, e.g., speculative execution (Brasser et al., 2017; Götzfried et al., 2017; Bulck et al., 2018; Gamaarachchi and Ganegoda, 2018; Van Bulck et al., 2020), will depend on CPU manufacturers' firmware fixes and thus are out of the scope of this study.

The threat model outlined above is more powerful and provides richer semantics of protection compared to the previous study (Sharma et al., 2019).

### 3.2 TEE-Graph architecture

We aimed to design an efficient confidential graph analysis framework. One of the key ideas of our framework is to leverage trusted execution environment (TEE) as a trusted computing base (TCB) to guarantee the confidentiality and integrity of the graph analysis in the cloud. We show that TEE can enable stronger and richer privacy protection and much more efficient solutions compared to existing pure-software cryptographic approaches (Sharma et al., 2019).

Figure 2 shows the core components and the workflow of the TEE-Graph framework. In the following, we describe the detailed role of each party in the collaborative computing scenario.

- **Contributors (C)**. Before participating in the collaboration, each contributor (*C*_*i*_) agrees on the data owner's privacy policy and the usage of their data and receives a unique ID. The contributor *C*_*i*_ first encrypts their portion of the graph *A*_*i*_, e.g., *C*_*i*_'s adjacency edges mapped to the *i*th row of the adjacency matrix *A*. Then, *C*_*i*_ performs remote attestation with the data submission service running in the cloud's TEE to ensure the correctness of the hardware and the trusted binary running in the cloud. It also conducts the Diffie-Hellman Key Exchange (DHKE) protocol to create a secure channel between TEE and the *C*_*i*_'s system. *C*_*i*_ sends *A*_*i*_ and other metadata via the secure channel. The contributor may continuously update their data, leading to an evolving graph. However, we focus on the simpler case of a snapshot graph in this study.
- **Cloud Provider (P)**. It hosts all the required components of TEE-Graph, including storage, TEE infrastructure, and trusted and untrusted codes. After instantiation, the TEE-based data submission service engages with each *C*_*i*_ and establishes secured channels via remote attestation. After receiving *A*_*i*_ along with other meta data, the service stores encrypted data in untrusted memory (disk) with ensured confidentiality and integrity. Another TEE-based service, i.e., spectral analysis service receives requests from the data owner and performs on-demand graph analytics confidentially.
- **Data Owner (O)**. After all the *C*_*i*_ submit their data, *O* requests to conduct spectral analysis on the collected graph data. *O* also performs a remote attestation with the spectral analysis service and establishes a secure connection for receiving the result.

### 3.3 Technical challenges

With the threat model and each party's role, we need to address three technical challenges in the TEE-graph design.

- **Ownership and Confidentiality Protection**. In a cloud-based environment, when data are submitted to the cloud, both the data contributors and the data owner lose control of the data as the cloud provider has the full control of the data. Our goal is to safeguard the privacy and ownership of the submitted data so that no other parties, including malicious contributors or the cloud provider, can infer private information or steal the data. With privacy regulations such as GDPR, data contributors should also be able to revoke sharing their private data at any time, without the concern that dishonest data owners will continue to use their data. Similarly, data owner wants to preserve confidentiality and ownership of the collected data to conform the privacy law.
- **Processing Big Graph with TEE restrictions**. Trusted execution environments (TEEs) have limitations, such as memory constraints, computational overhead, and interactions with untrusted parts of the system. We aim to address these challenges, specifically for the Lanczos and Nystrom methods.
- **Protecting TEE Access Patterns**. As explained in Section 2, TEE is still vulnerable to access-pattern-based side-channel attacks, which are difficult to mitigate. We will thoroughly examine the TEE-Graph framework, identify data-dependent access patterns, and create efficient obfuscation mechanisms to mitigate the issue.

## 4 Technical details

We design the following solutions to address the three technical challenges.

### 4.1 Maintaining ownership and confidentiality of graph data

We leverage TEE to manage the ownership of both contributor and data owner's ownership on the data even after submitting the data to untrusted cloud. We designed the TEE-Graph to ensure that only the TEE has access to private data. Therefore, no other party, including the cloud provider, can learn any sensitive information during processing or while at rest. Before we describe the unique feature, we give the graph encoding method used in our approach.

#### 4.1.1 Graph encoding

Large graphs are often sparse. We use a simple sparse encoding method for the graph adjacency list of each contributor. This method assigns a unique identifier to each vertex and each vertex (contributor) maintains a list of the neighboring vertices. Let us consider a typical graph matrix for spectral analysis: the normalized Laplacian graph matrix *L* = *I*−*D*^−1^*W* (the definition in Section 2.1). It is clear to infer that *L* is still sparse if *W* is sparse. In sparse encoding, the zero entries are skipped, while the non-zero ones are encoded as (*i, j, v*) for entry index (*i, j*).

Each contributor will submit a sparse row of the matrix, corresponding to the corresponding node's outlinks in the graph. Each contributor will also have a public key pair for signing their submitted data, and the TEE maintains a public key database for verification. With an established secure channel, a contributor signs the list of their neighbors with a digital signature, *S*_*i*_, and submits the graph data to the submission service. The contributor needs to keep the signature *S*_*i*_ for possible later revocation.

We used the 128-bit AES-CTR encryption mode to encrypt the received list with a TEE-specific key, which will be discussed later, and stored it in a block file. Due to AES, it does not increase the ciphertext from the original plaintext data, a significant benefit compared to the non-TEE approaches that depend on expensive homomorphic encryptions (Sharma et al., 2019).

#### 4.1.2 TEE-specific key

We implement the ownership and confidentiality protection by leveraging a key feature of TEE: the TEE-specific private key. Specifically, the TEE can generate a TEE-specific private key that only the TEE can access, inaccessible even to the TEE owner. Let *K*^*TEE*^ denote such a private key, which will be used to encrypt the graph data stored in the cloud. *K*^*TEE*^ is encrypted and stored on the disk with the sealing key that is bound to the TEE binary and its collaterals, provided by the manufacturer. *K*^*TEE*^ can only be restored and decrypted when the same TEE wants to read it; otherwise, no one can ever restore the key. This TEE-specific private key management is a unique feature of TEE and has been extensively used in practice (Karande et al., 2017; Van Schaik et al., 2020). After instantiation of TEE-Graph, the submission service TEE generates *K*^*TEE*^. *K*^*TEE*^ is shared between the system services and never gets out of the TEE. After receiving the data *A*_*i*_ from the contributor *C*_*i*_, the submission service encrypts it with *K*^*TEE*^ and store the encrypted data on the cloud storage.

We do not aim to hide each contributor's submission activity, which is also impossible. The sensitive part of the submission is the specific edges and the node degree, e.g., who the contributor interacts with and how many such neighbors are in the graph. Section 4.3 discusses details about access-pattern protection in the submission process and the spectral analysis.

The benefit of this procedure is two-fold. (1) The data owner can only use the collected data for the specific graph analysis service, which cannot be moved for other uses. It enforces the agreement between the contributors and the data owner. A relaxed access model can also be easily implemented on top of that, e.g., the data owner fully owns a specific contributor's data, i.e., with an owner provided *K*^*TEE*^. (2) Since the submitted data cannot be moved for other uses, we allow a certain submitted data item to be removed securely, guaranteeing the contributor's right of revocation. Thus, this procedure guarantees the ownership and confidentiality of data for both the contributors and the data owner.

#### 4.1.3 Revocation

The contributor can submit the signature of the specific submission (with a specific submission ID) they want to revoke to the revocation service. The service will verify the signature with the stored *C*_*i*_'s public key and delete the requested item. It does need to record the information (contributor ID and submission ID) together with the public key database to implement this revocation operation. However, this cost is linear to the number of contributors and highly acceptable. The access patterns of the revocation operation do not reveal any private information in the graph.

### 4.2 Handling big graphs with TEE

Processing large graphs in TEE presents multiple challenges that must be addressed. One of the primary concerns is maintaining the integrity of graph data throughout the lifecycle of TEE-Graph. This necessitates implementing robust security measures to prevent unauthorized access and tampering. Additionally, the limited TEE memory for some TEEs, e.g., Intel SGX, poses a challenge in efficiently processing vast amounts of data. In the following section, we will outline the components of TEE-Graph and explain how they work together to efficiently manage and analyze big graphs with TEEs.

**Figure 4:**
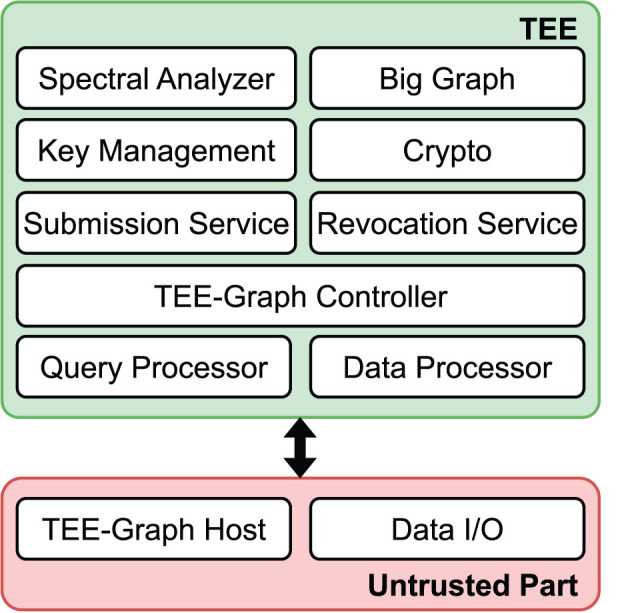
Overview of the TEE-Graph Components.

**Components of TEE-Graph**. We design TEE-Graph based on the most popular TEE, Intel SGX. Intel SGX requires the TEE application divided into to the trusted and untrusted parts. Figure 4 shows the components of the framework. The untrusted part only performs the initiation of TEE-Graph along with basic read/write operations. The trusted part performs the core operations, including the graph submission service and the spectral analysis service.

- **Untrusted Part**. Since Intel SGX depends on untrusted memory for network and IO operations, our design needs to address the confidentiality of the data that depends on the untrusted part. Therefore, our design involved minimal tasks that relied on the untrusted part of the framework. The untrusted part of the TEE-Graph instantiated the framework. The remote attestation procedure guarantees that the untrusted part honestly loaded the correct binary in TEE to start the services in the enclave. Second, the IO operations, i.e., read/write data from TEE, depend on the untrusted part of the framework. We maintain privacy-preserving data read/write operations to hide all the sensitive information in processing the graph.
- **Trusted Part**. Major components of the TEE-Graph remain in the trusted part. The key management module maintains the session key after each remote attestation performed by either the contributors or data owner. This module also securely generates the *K*_*TEE*_ with the manufacturer's crypto module so that no side-channel information is leaked. TEE-Graph's crypto module performs all the encryption/decryption of the submitted data, intermediate sensitive data, and spectral analysis results. The Big Graph module maintains the Big Graph's read/write operations and on-demand loading of subgraphs. The spectral analyzer module performs the spectral analysis algorithms upon the data owner's request. This module interacts with the Big Graph module and performs the required operations, e.g., sparse matrix-vector multiplication. The result is encrypted with the data owner's private key and returned to the data owner.

#### 4.2.1 Integrity guarantee

We maintain the integrity of the graph data both in transit, at rest, and during computation. While TEE assures the integrity of enclave memory, data residing in untrusted memory remain vulnerable and can be modified. The integrity of the data transferred from the untrusted part will be verified inside the enclave.

We consider three possible attacks to integrity: (1) modify a data block, (2) shuffle a block with another block in the same file, and (3) insert a block from a different file (or a phase's output that is encrypted with the same key). To address all the attacks, we include the following attributes in the block: (i) Block ID, so that block shuffling can be identified, (ii) File Id, so that no block from different files can be inserted, and (iii) the block-level message authentication code (MAC). At the end of each block, a MAC is attached to guarantee the integrity of records, before the whole block is encrypted. We also use the randomized encryption mode AES-CTR to make sure identical blocks will be encrypted to non-distinguishable ones so that adversaries cannot trace the generated results in the TEE-Graph's workflow. A simple verification program runs inside the enclave that verifies the IDs and MAC after reading and decrypting a block.

#### 4.2.2 Efficiency

Some TEEs, i.e., Intel SGX, may have a very limited TEE memory size. Designing TEE applications that use big graphs can be quite challenging due to the limited memory of TEE. It is important to consider the memory constraints when developing such applications to ensure they run efficiently.

**Figure 5:**
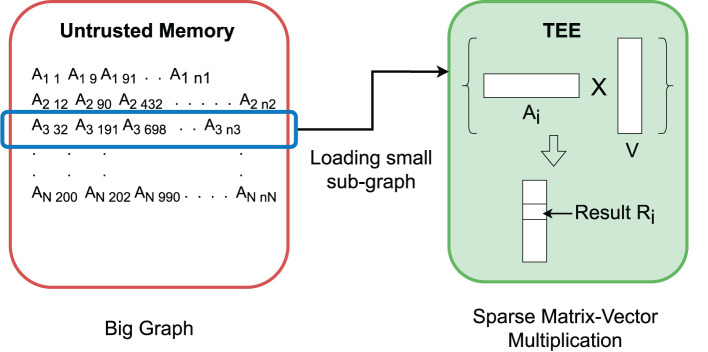
Sparse matrix-vector multiplication for big graphs in a small trusted computing base (TCB). TEE loads a small sub-graph, perform sparse multiplication with the corresponding vector, and stores the positional value in the resultant vector.

The main memory consumption for performing spectral analysis is large for large graphs. To address the TEE memory limitation, we use a stream processing method to process the graph: only the requested part is processed in the enclave while the rest of the graph remains in the encrypted form in the untrusted memory. The core operation of the Lanczos method can be conveniently converted to stream processing. Specifically, sparse matrix-vector multiplication is streamlined by loading each row of the sparse matrix sequentially (Figure 5). This also allows a big graph to be partitioned and processed in parallel in multiple TEE threads. In contrast, the Nystrom method depends on sampling to reduce the large graph to a manageable size subgraph. Note that these activities will not breach the private information embedded in the row if the access pattern protection method is applied (details in Section 4.3).

The following methods can be used to further improve the performance.

- **Multi-threaded enclaves**. In sparse matrix-vector multiplication, each partition of the graph can also be processed in parallel with multi-threaded enclaves. Most TEE implementations such as SGX also enable multi-thread confidential processing.
- **GPU TEE**. GPUs can significantly speed up large matrix computation. However, when the task is moved out of the TEE to a GPU, the security is not guaranteed. While several methods (Hunt et al., 2020; Deng et al., 2022; Yudha et al., 2022) have tried to incorporate GPUs in TEE-based confidential computing, the performance gains of using GPUs in these methods are quite limited. Nvidia recently launched GPU TEE (e.g., H100) to enable TEE features at the hardware level, which will be tested in our future work.

### 4.3 Access-pattern protection

One of the most challenging aspects of a TEE framework is safeguarding data access patterns. We have carefully examined the TEE-Graph framework, starting from contributors' graph submission to the data owner's receipt of the results and developed measures to protect these access patterns.

**Figure 6:**
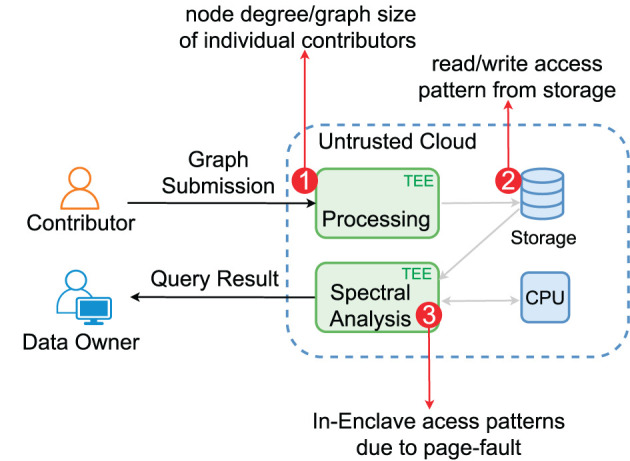
Identifying access pattern vulnerability in TEE-Graph.

Note that we do not aim to hide which contributor submitted which row. However, we try to protect the content (or the access patterns) of the row elements, which breaches edge privacy and node privacy. Figure 6 depicts the access points where access patterns need to be protected. (1) During contributors' graph submission, the untrusted part of the submission service can infer a contributor *C*_*i*_'s node degree via the length of the submitted data even though the data are fully encrypted. The node degree might be used, e.g., in breaching the identity of *C*_*i*_. (2) During graph processing, each row of the matrix is loaded, again, the length of which can be monitored to review the node degree of *C*_*i*_. (3) Finally, within the spectral analysis enclave, adversaries via the compromised OS can observe the in-enclave page-level access patterns to infer the node degree information and the indices of the non-zero elements, which expose the topology of the graph.

**Algorithm-specific memory access pattern**. We have also conducted a thorough analysis of the spectral analysis algorithms utilized in both Lanczos and Nystrom methods (the detailed steps are in Algorithms 1, 2 in Section 2).

**Figure 7:**
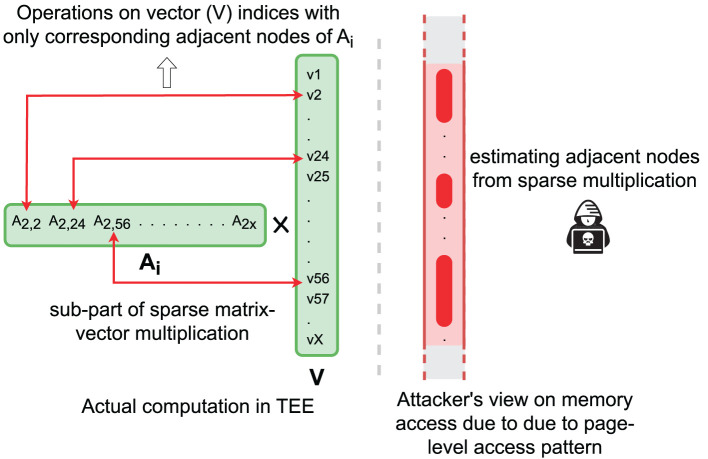
Attacker estimates the neighboring nodes from memory page-level side-channel attack.

During the computation of sparse matrix-vector multiplication in the Lanczos method, we load each matrix row *E*(*A*_*i*_) sequentially in TEE and decrypt it. Note that the sparse encoding only keeps the non-zero entries of the matrix row, and thus, the corresponding positions in the vector will be revealed, which can be used to infer the edge. By monitoring this information, the adversary can recover the graph topology. Figure 7 shows the access pattern and attacker's view on the access patterns. Such an attack completely breaches the confidentiality of graph structure during graph analysis.

The Nystrom method uses random sampling in the first stage, where selecting or not selecting a node seems non-sensitive information. However, for each selected row, the selected columns need to be extracted, which again reveals the topology of the sampled subgraph. Furthermore, the last step of Nystrom method also accesses the complete columns of the graph matrix, which also expose partial graph topology.

Compared to these operations, the eigen-decomposition of the small matrix in these methods is less sensitive once the proposed protection approach is applied.

#### 4.3.1 Differentially private edge injection to protect access patterns

Previously, we have proposed the bin-based localized differentially private data submission method to disguise the non-zero entries (Sharma et al., 2019). We have found that this algorithm can also address the access pattern issues with the TEE-based processing. To make it self-contained, we will briefly describe the bin-based graph perturbation first and analyze how it protects access patterns during spectral analysis.

Via the previous analysis, we have known that without protection, the adversary can infer node degrees during data submission and further infer the indices of non-zero entries in spectral analysis. The basic idea is to inject fake non-zero entries, i.e., encrypted zero entries, but the adversary cannot distinguish them from them in the encrypted form. Apparently, a trade-off exists: the more the zero entries, the more the submission, storage, and computation costs.

We turn to an efficient approach based on differential privacy (Dwork, 2006). In the standard differential privacy definition, the goal is to disguise any specific person among the entire set of persons that are related to the database. Thus, the key factor, the sensitivity of function, is applied to the whole dataset, which, however, results in very large sensitivity for functions related to node degree on graph datasets. As a result, data contributors have to add many fake items to achieve the desired differential privacy, which seriously impairs sparsity. Specifically, let the query function *F*() about node degree, say finding the node degree ranked at *k*. Let *A* and *A*′ be the *neighboring graphs* which differ by only one node. Thus, the sensitivity Δ = max{*F*(*A*)−*F*(*A*′)} is the difference between the largest and the smallest node degree. For a graph of *N* nodes, this sensitivity can be up to *N*.

To achieve a better balance between privacy and sparsity, we use a bin-based method to achieve weaker contributor indistinguishability, which is reduced from the whole graph to a subset of nodes in a bin. Specifically, we sort the nodes by their node degrees and then partition the degree distribution by bins. The contributors in the same bin select the number of fake edges with the bin-specific parameter, where the function sensitivity can be much smaller. The node degree distribution can be estimated with the node degrees of randomly sampled nodes. This can be achieved by the data owner asking some randomly selected data contributors to submit encrypted node degrees before them submitting the graph data. The data owner can then build a histogram to approximate the node degree distribution. Apparently, this additional cost is quite low.

The method will generate an equi-height histogram with the sample node degrees, e.g., for a 100-bin histogram, each bin contains approximately 1% of the nodes. The number of bins is chosen so that each bin contains a moderate number of nodes, for example, a value in (50, 100) to provide satisfactory indistinguishability. Let *U*_*i*_ be the maximum node degree in the *i*-th bin, and *L*_*i*_ be the minimum degree in the *i*-th bin. Now let *A* and *A*′ be the neighboring graphs which differ from each other by only one node *in the bin*. We can derive the sensitivity $\Delta_{i}=\max\left\{F\left(A\right)-F\left(A^{\prime }\right)\right\}=U_{i}-L_{i}$, which should be much smaller than *N*.

According to the noise design of differential privacy, we derive that the parameter *b* of Laplace distribution *Lap*(0, *b*) to be (*U*_*i*_−*L*_*i*_)/ϵ. However, this noise can be negative, which does not make sense, e.g., asking the contributor to remove real edges and thus destroying the authenticity of data. To avoid this problem, we add an offset to the noise to make it positive, which reduces the overall sparsity but still satisfactorily preserves both privacy and authenticity. For a specific *b*, we can always identify the bound *p* for *Pr*(*x* < *p*) <= 0.01 (*p*≈−3.912 for *b* = 1 and *p* linearly scales with *b*: *p*≈−3.912*b*), i.e., if we add an offset |*p*| to the distribution, we can make sure the majority of population (>99%) positive. With such an offset, the number of fake edges, the number of fake edges, *k*_*i, j*_, is chosen as follows:


$$\begin{array}{l} k_{i,j}=|p_{i}|+\delta_{i,j}, \end{array}$$

where |*p*_*i*_| is the offset and δ_*i, j*_ is a random integer drawn from Laplace(0, (*U*_*i*_−*L*_*i*_)/ϵ) to make *k*_*i, j*_>0. With such a noise design, the nodes in the same bin satisfy ϵ-differential privacy on node-degree based functions.

By preserving node-degree differential privacy, edge differential privacy is also satisfied. We define *A* and *A*′ as a pair of neighboring graphs, if they only differ by one edge. The problem of checking the existence of an edge can be transformed to an edge counting query function. Let us look at any arbitrary edge counting functions. Clearly, the sensitivity of such a function is 1. Thus, Laplace(0, 1/ϵ) is used to generate the noisy edges. Since the parameter (*U*_*i*_−*L*_*i*_)/ϵ used for disguising node degrees is no less than 1/ϵ, the fake links generated for protecting the privacy of node degrees also protect edge privacy.

Algorithm 3 gives the details of our privacy preserving sparse submission algorithm.

**Algorithm 3:**
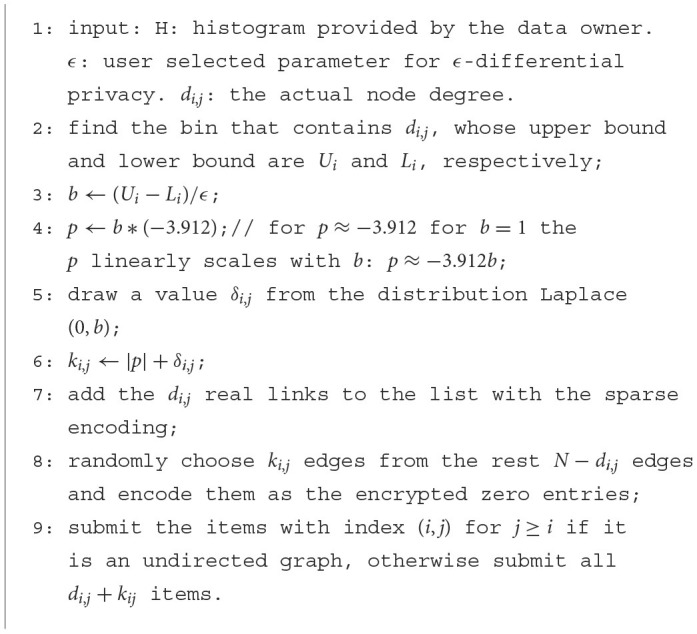
Privacy preserving sparse submission (H, *∈*, *d_i,j_*).

The benefit of random fake edges also extends to the access-pattern protection during spectral analysis. Specifically, when doing matrix-vector multiplication, the fake edges are loaded as normal ones and participate in the computation. The adversary does not obtain additional information by observing these access patterns. However, since their values are zero, the final computational result does not change. We have observed that the increased costs by using the differentially private fake edge injection are small.

## 5 Experimental evaluation

The evaluation will verify that (1) the TEE-Graph approach can significantly reduce the costs of confidential graph spectral analysis, compared to existing pure software cryptographic approaches, and (2) with our unique design and a small extra cost, it can also effectively disguise access patterns and thus protect from many TEE side-channel attacks.

Note that the proposed framework can adopt the exact Lanczos and Nystrom algorithms and execute them just like in plaintext computation. Our previous study (Sharma et al., 2019) has evaluated the utility of these approximate spectral analysis algorithms. Thus, we do not repeat this experiment here.

### 5.1 Experiment setup

#### 5.1.1 Implementation

We have developed the major components of the TEE-Graph framework with C++ and the Intel SGX SDK for Linux. The framework operates entirely within the enclave, except for a small part located outside the trusted area that handles block-level read/write requests from within the enclave. To encrypt data blocks in the untrusted memory, we use 128-bit AES-CTR encryption. Our implementation of Lanczos and Nystrom methods is designed to work within the functional limitations of TEEs. We utilize the C++ implementation of the Eigen library for eigendecomposition of small matrices in both methods. Additionally, we apply graph perturbation technique using differential privacy to safeguard graph submission information and in-enclave access patterns.

For comparisons with state-of-the-art crypto approaches, we utilize the PrivateGraph methods developed in our previous work (Sharma et al., 2019). Its Paillier method was implemented with C++ using GMP big integer library and Armadillo linear algebra library, and used the 80-bit security setup with 10 fractional-digit precision for floating-integer conversion. The HELib library (github.com/shaih/HElib) was used for the RLWE scheme with 32-bit plaintext encoding and the ciphertext packing technique (Smart and Vercauteren, 2012). Note that these settings are theoretically weaker than 128-bit AES-CTR we used for TEE-Graph in security guarantee.

Our experiments were performed on a Linux machine equipped with an Intel(R) Core(TM) i7-8700K CPU of 3.70 GHz processor and 16 GB of DRAM.

#### 5.1.2 Benchmark measures

To benchmark and compare the Lanczos and Nystrom methods, we measure the communication costs for both the data contributors and the data owner for all compared methods. We also analyze the storage and computation costs on the cloud side for major operations in spectral analysis for different methods.

#### 5.1.3 Datasets

To make the results comparable, we adopted the same three graph datasets in the SNAP database (snap.stanford.edu) that were used by pure-software cryptographic approaches (Sharma et al., 2019). They were originally used to study social circles in the three popular social networks—Facebook, Twitter, and GPlus. We make the edges undirected for easier processing in the evaluation. Table 1 describes the properties of the graph data we used in the benchmark.

**Table 1:** Describes the properties of the datasets used in the benchmark.

**Datasets**	**Nodes**	**Edges**	**Size**
Facebook	4039	88234	854 KB
Twitter	81306	1768149	44 MB
Gplus	107614	13673453	1.34GB

### 5.2 Result analysis

All the costs in the following discussion are based on the differentially private graph element submission conducted by data contributors. The next section will focus on the optimal parameter setting and extra costs associated with the differentially private submission method.

#### 5.2.1 Contributor's cost

In Table 2, you can find the average cost for contributors in different methods, including the encryption cost and the amount data to be uploaded. The baseline PrivateGraph methods Paillier and RLWE use additive homomorphic and somewhat homomorphic encryption methods, respectively, which incur significantly higher costs than the AES encryption used in TEE-Graph. TEE-Graph uses a compressed encoding and block-based encryption method, with a fixed block size of 4KB. This method does not require element-wise encoding or complex cryptographic protocols. Furthermore, AES does not increase the cipher text size, which is significant for the data to be transferred to and stored in the cloud. Therefore, contributors can benefit from significantly lower encryption and data submission costs for TEE-Graph than those for Paillier and RLWE.

**Table 2:** Contributor's average cost for sparse submission.

**Method**	**Encrypt** ***A***_*****i*****_ **(s)**			**Upload E(** ***A***_*****i*****_ **) (MB)**		
	**FB**	**Twitter**	**GPlus**	**FB**	**Twitter**	**GPlus**
Paillier	0.04	0.03	0.22	0.006	0.005	0.032
RLWE	0.64	0.51	3.28	12.1	9.6	61.9
TEE-Graph	0.00005	0.00003	0.00009	0.0001	0.0002	0.001

#### 5.2.2 Data owner's costs

In PrivateGraph methods, the data owner needs to actively participate in the iterative process of spectral analysis, which incurs significant costs. For example, in each iteration of the Lanczos process with the additive homomorphic encryption (AHE)-based implementation, the data owner needs to mask/demask the vector in confidential matrix-vector computation. In the Nystrom method, the data owner needs to conduct the small matrix decomposition locally. In contrast, TEE-Graph has everything done within the TEE in the cloud and the data owner takes almost zero cost in the process of spectral analysis (except for submitting the service request and receiving the result). For the experiment, we used 30 iterations and 10 clusters. Table 3 displays the comparison of the accumulated costs of encryption/decryption and communication for all methods. Again, we see TEE-Graph has huge cost advantages for the data owner.

**Table 3:** Data owner's costs for confidential Lanczos methods.

**Method**	**Encryption cost (s)**			**Communication cost (MB)**		
	**FB**	**Twitter**	**GPlus**	**FB**	**Twitter**	**GPlus**
Paillier	696	12,809	17,969	108	1,983	2,789
RLWE	30	2,915	443	2,201	2,915	4,673
TEE-Graph	~ 0	~ 0	~ 0	~ 0	~ 0	~ 0

#### 5.2.3 Cloud storage cost

All these methods request the encrypted data stored in the cloud for applying different analytics algorithms or evolving graph analysis. The cloud storage cost is approximately the sum of all Contributors' submitted data. Recall that both Paillier and RLWE encryption methods require elementwise encryption. Additionally, due to constraints on the number of elements per ciphertext in RLWE, these methods require significantly larger storage costs. In TEE-Graph, we achieve much lower costs through block-wise AES encryption that results in the approximately same size of the plaintext data. Table 4 demonstrates the comparison on cloud storage costs for all the methods.

**Table 4:** Cloud storage costs with sparse submission.

**Method**	**Facebook**	**Twitter**	**GPlus**
Paillier	24.4 MB	372.9 MB	3.2 GB
RLWE	47.8 GB	729.8 GB	6.3 TB
TEE-Graph	845 KB	44.6 MB	1.35 GB

KB, kilobytes; MB, megabytes; GB, gigabytes; TB, terabytes.

#### 5.2.4 Cloud computational cost

**Figure 8:**
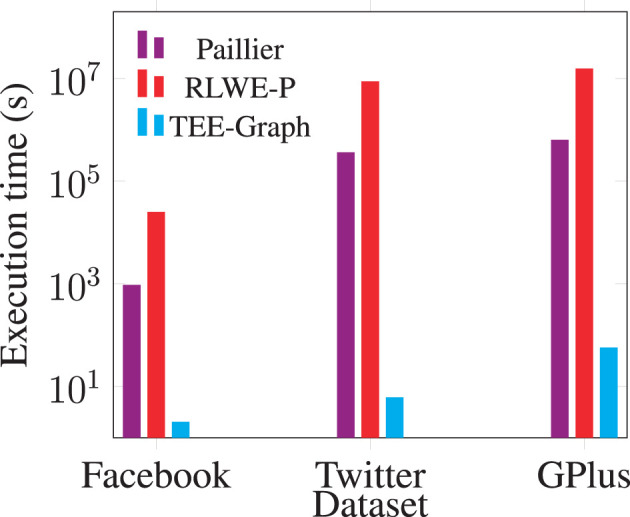
Comparing cloud cost for matrix-vector multiplication.

Figure 8 shows the comparison of computational cost for running the spectral analysis algorithms. We take the most representative operation: computing one matrix-vector multiplication confidentially for comparison, which is used in both Lanczos and Nystrom methods. All the pure-software implementations demand expensive homomorphic operations over encrypted data, which incurs much higher costs than decrypting AES-encrypted data and computing with plaintext data inside the TEE. Our experiment shows that TEE-Graph performs 6,000 × to 150,000 × faster than baseline methods on the core operation.

### 5.3 Cost of access pattern protection

In Section 3, we have analyzed the threats caused by exposing the access patterns in the data submission and spectral analysis computation stages. To address this vulnerability, we developed the differentially private method to inject fake encrypted edges (with zero weight value) in data submission. The fake edges can effectively protect the confidentiality of access patterns as well in the spectral analysis stage. In the following, we will show the additional costs brought by this technique.

#### 5.3.1 Graph perturbation cost

In the sparse format, the element will be encoded as (*i, j, v*). The PrivateGraph methods have to keep (*i, j*) in plaintext, but encrypt *v*; in contrast, TEE-Graph packs the entire entry in a block and encrypts the entire block. Although the index is not revealed, but the size of the block and the element-wise access patterns can still reveal the confidential graph information. Section 4.3.1 presented the differential privacy based method to determine the random number of zero elements to be added to the submitted rows of the graph matrix. The total number of submitted elements depends on the personalized privacy parameter ϵ. We select the number of bins so that the number of nodes in each bin is in [50, 100] to provide sufficient indistinguishability within the bin. With a well-accepted privacy setting ϵ = 1.0, we have the results in Table 5. The numbers in the column “|*E*| pert.” are the average of 10 runs. Apparently, the increase of the total number of edges is quite acceptable.

**Table 5:** Perturbation parameters and results.

**Dataset**	**nbins**	**nodes/ bin**	**orig. |*E*|**	**pert. |*E*|**	**% inc**.
Facebook	100	40	84243	99965	18.66
Twitter	1,000	76	1242390	1527286	22.93
GPlus	2,000	52	12113501	13228599	9.21

“orig. |*E*|”, the number of original edges. “pert. |*E*|”, the number of edges after perturbation. “%inc.”, percentage of increase.

#### 5.3.2 Additional computational costs

**Figure 9:**
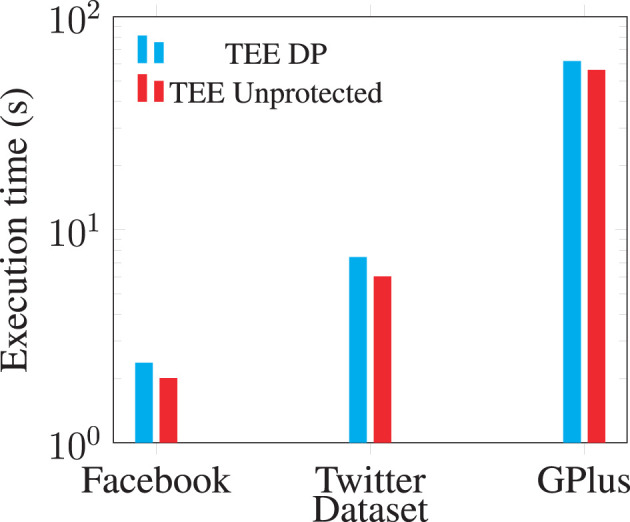
Access-pattern protection cost for sparse matrix-vector multiplication in TEE-Graph.

With the added zero-weight edges, we evaluate the additional costs in the spectral analysis. We use the core operation: sparse matrix-vector multiplication, to show the performance impact on TEE-Graph. Figure 9 shows the overhead for access pattern protection is quite limited: about 10–25% of the overall cost.

## 6 Related work

### 6.1 Cryptographic approaches

Most software approaches for confidential data mining utilize homomorphic encryption, e.g., RLWE (Brakerski, 2012), or secure multi-party computation (Yao, 1986; Huang et al., 2011; Mohassel and Zhang, 2017), or combinations of these primitives. However, the high communication overhead and execution time make them are still impractical for larger datasets and complex algorithms.

Our previous study on confidential graph spectral analysis: PrivateGraph (Sharma et al., 2019) employs a hybrid cryptographic protocol to engage the three parties: the data contributor, the data owner, and cloud provider to achieve the best performance for pure software based confidential graph spectral analysis so far. Additive homomorphic encryption and somewhat homomorphic encryption were used in different implementation schemes and a few novel ideas, e.g., masking/demasking vectors with the *Learning with Error* problem (Regev, 2005). Despite the application of novel ideas, the overall costs are still extremely high for all the three parties.

We show in our TEE-Graph approach that by using the hardware-assisted TEE approach, we are able to develop much more cost-effective solutions than the pure-software approaches. Furthermore, we can also offer richer privacy protections, e.g., guaranteeing data contributors' right of revocation for shared data.

### 6.2 TEE-based approaches

TEE-based privacy-preserving frameworks become popular in the last few years. Researchers have been exploring TEE-based applications for mainly (i) data-intensive analytics (Dinh et al., 2015; Schuster et al., 2015; Zheng et al., 2017; Alam et al., 2021) and (ii) data management (Priebe et al., 2018; Antonopoulos et al., 2020; Sun et al., 2021). VC3 (Schuster et al., 2015) and M2R (Dinh et al., 2015) extends and utilizes the Hadoop System, where the most sensitive part of the computation takes place in TEE. However, it still depends on a lot of untrusted data processing, which leaks information. Opaque (Zheng et al., 2017) tries to revise Spark for SGX. They focus on the data access patterns between computing nodes and illustrate how adversaries can use these to infer sensitive information in the encrypted data. Database systems such as ObliDB (Eskandarian and Zaharia, 2019) perform an extensive analysis on protecting user data during SQL operations. They provide a set of oblivious methods for everyday SQL operations. However, these methods have significant cost overhead compared to unprotected database systems. On the other hand, Enclage (Sun et al., 2021) and Always Encrypted (Antonopoulos et al., 2020) provide a practical notion for TEE-based databases which tried to give the balance between privacy and efficiencies where leaving most critical side-channel attacks, e.g., controlled channel attacks out of their scope, making it less secure compared to ObliDB.

While these frameworks have been designed for generic data, and some might be applied to graph analytics, no significant work has been done to address the specific challenges with confidential graph analytics using TEE, especially, the access-pattern protection. Du et al. (2023) proposed a graph encryption method focusing on the shortest distance query. While this approach hides nodes and edges using PRF, the graph's topology remains the same. Using multiple queries, adversaries can still observe the access pattern during graph processing, making the graph and query vulnerable to powerful adversaries. On the other hand, our approach provides a complete solution for protecting the contributors' and data owner's ownership control and access patterns during data submission and computation in the cloud.

### 6.3 TEE access-pattern protection

Data access-pattern protection has been a major approach to addressing many side-channel attacks. Our approach represents a task-specific more efficient protection method, specifically designed for graph spectral analysis. Data oblivious programming is a more generic solution to protect access patterns. It contains three major approaches: (1) Manually constructing solutions with data oblivious primitives, such as ORAM for disguising data block accesses (Sasy et al., 2018), CMOV instructions for disguising branching statements (Ohrimenko et al., 2016) and specific data oblivious algorithms (Batcher, 1968; Krastnikov et al., 2020). However, it requires developers to re-examine every statement of the program and revise with corresponding data oblivious method, which is expensive and error prone. (2) Automated conversion approaches, such as the circuit-based conversion (Büscher et al., 2018; Ozdemir et al., 2022) or special compilers (Liu et al., 2015; Rane et al., 2015) are still not mature. (3) Semi-automated approaches, e.g., the framework-based SGX-MR (Alam et al., 2021), might be a promising direction that hides the protection measures in a framework such as MapReduce and the developer only needs to handle much smaller and simpler pieces of code and access patterns. There has been an extensive study for comparing these approaches (Alam and Chen, 2023).

### 6.4 Application of differential privacy in graph analysis

Privacy-preserving graph data publishing (Zhou et al., 2008) is somewhat related to confidential graph data mining. However, it differs from our approach by using a different threat model: graph data publishing does not assume proprietary data sharing but sharing data with the public. Thus, the adversaries will be among potential data miners. Consequently, differential privacy has been applied to perturb the published graphs so that the privacy of the individuals associated with the graph is properly protected (Kasiviswanathan et al., 2013; Wang et al., 2013). Such perturbed graphs can approximately preserve the information of the original graph.

In a decentralized setting, local differential privacy (Duchi et al., 2013) is a widely used technique that enables researchers to safeguard data privacy without relying on a service provider. The approach works by introducing random perturbations to data locally before transmitting it to an untrusted service provider or data owner. Real-world applications, such as Google Chrome (Erlingsson et al., 2014) and macOS (Wang et al., 2017), have already incorporated this method. However, when using local differential privacy to collect graph data, employing randomized responses (Warner, 1965; Du et al., 2023) to perturb each element of the graph can result in excessive noise. To address this issue, Qin et al. (2017) and Ye et al. (2020) proposed collecting the graph structure in the form of degree vectors, which represent the neighbors of the graph. In this technique, the service provider initially divides all the nodes into k non-overlapping subsets. Instead of using the actual connections of a node *u*, the degree vector of *u* represents the number of connections *u* has for each subset. Finally, noise is added to the degree vector to perturb the number of connections. While these approaches reduce the overall noise of the graph, they still significantly modify the actual structure of the graph.

In contrast, our objective is not to divulge graph structures but to use randomization to inject zero-weight edges to disguise sensitive information such as node degrees and access patterns. The encrypted edges are not distinguishable, and the zero-weight edges do not change the computational results.

## 7 Conclusion and future work

With big graphs collected, stored, and analyzed in the cloud, data confidentiality and ownership are becoming an increasingly concerned issue. Most recent studies on confidential graph analysis have been focused on software cryptographic approaches. Only a few studies are based on trusted execution environments (TEEs), which have not sufficiently addressed the two critical issues: data contributors' ownership and access-pattern protection. We study the problem of confidential graph spectral analysis for large graph data in the cloud and design the TEE-Graph approach to address these critical issues. Our experimental results show that TEE-Graph performs much faster than software cryptographic approaches with additional benefits in ownership and access-pattern protection.

The future work may include the following directions. (1) Extend the analysis part to more tasks for big graphs and protecting their access patterns efficiently. (2) Consider integrating more convenient access-pattern protection frameworks, such as SGX-MR (Alam et al., 2021). (3) Study the unique problems with evolving graphs, e.g., access patterns and ownership protection.

## Data availability statement

Publicly available datasets were analyzed in this study. This data can be found at: https://snap.stanford.edu/data/index.html#socnets.

## Author contributions

AA: Conceptualization, Data curation, Formal analysis, Investigation, Methodology, Software, Validation, Visualization, Writing—original draft. KC: Conceptualization, Formal analysis, Funding acquisition, Methodology, Project administration, Resources, Supervision, Writing—review & editing.

## Footnotes

^1^In this paper, “data privacy” and “data confidentiality” are used interchangeably.
